# Use of Personal Care Products and Semen Quality: A Cross-Sectional Study in Young Danish Men

**DOI:** 10.3390/toxics8030062

**Published:** 2020-08-22

**Authors:** Kajsa Ugelvig Petersen, Ahmad Mahmoud Balkiss, Katia Keglberg Hærvig, Jens Peter Ellekilde Bonde, Karin Sørig Hougaard, Gunnar Toft, Cecilia Høst Ramlau-Hansen, Sandra Søgaard Tøttenborg

**Affiliations:** 1 Department of Occupational and Environmental Medicine, Bispebjerg and Frederiksberg Hospital, University of Copenhagen, Bispebjerg Bakke 23, 2400 Copenhagen NV, Denmark; ahmad.balkiss@gmail.com (A.M.B.); katia.keglberg.haervig@regionh.dk (K.K.H.); Jens.Peter.Ellekilde.Bonde@regionh.dk (J.P.E.B.); sandra.soegaard.toettenborg@regionh.dk (S.S.T.); 2 Department of Public Health, Faculty of Health and Medical Sciences, University of Copenhagen, Øster Farimagsgade 5, 1014 Copenhagen K, Denmark; KSH@nfa.dk; 3 National Research Centre for the Working Environment, Lersø Parkallé 105, 2100 Copenhagen Ø, Denmark; 4 Department of Clinical Epidemiology, Aarhus University Hospital, Olof Palmes Allé 43-45, 8200 Aarhus N, Denmark; gunnar.toft@au.dk; 5 Department of Public Health, Research Unit for Epidemiology, Aarhus University, Bartholins Allé 2, 8000 Aarhus C, Denmark; chrh@ph.au.dk

**Keywords:** personal care products, cosmetics, semen quality, male infertility, reproductive health, cohort, epidemiology

## Abstract

Personal care products (PCPs) may contain multiple chemicals capable of harming male reproductive function. The aim of this study was, therefore, to assess aggregated PCP exposure and potential associations with measures of semen quality in young men. Participants (*n* = 1058, age 18–21) were sampled among young men from the Danish National Birth Cohort (DNBC). Upon recruitment in 2017–2019, each man answered an online questionnaire and provided a semen sample. Exposure to 12 common types of PCPs was derived from the questionnaire, and the extent of use and co-use was analyzed. We applied a negative binomial regression model to estimate percentage differences in semen parameters between low, medium and high PCP exposure groups. All participants were exposed to at least one PCP more than once a week, resulting in a mean number (SD) of 5.3 (2.0) PCPs currently used. Most participants (92%) were also exposed to fragranced products on a weekly basis. Little association was observed between aggregated exposure to PCPs and sperm concentration, total sperm count, semen volume, sperm motility and morphology. Despite prevalent use of multiple PCPs, we found little indication of adverse effects of aggregated overall or fragranced PCP exposure on semen quality.

## 1. Introduction

Men burdened by infertility is a global health concern [1]. The psychological, social and economic consequences of a diminished capacity to father children are often severe and range beyond individuals to whole families and society at large. While considerable differences in male reproductive health exist within and between developed countries, a negative trend of low semen quality and high incidence of cryptorchidism, hypospadias and testicular cancer has been observed in many areas [2,3]. Thus, 35% of young men in Denmark have low semen quality [4]. Both genetic and environmental factors may contribute to this deficit in reproductive health [3]. At present, several common consumer products invented to ease our everyday lives are suspected of impairing key reproductive functions [5].

Personal care products (PCPs) include all non-pharmaceutical items consumed or applied to enhance personal health, hygiene or appearance [6]. While these products typically contain a multitude of chemicals, common ingredients include phthalate esters, parabens, ultraviolet (UV) filters, polycyclic musks, antimicrobials, formaldehyde and formaldehyde-releasers [5,7]. In addition, non-intentional, technically unavoidable contamination with metals such as lead, cadmium, antimony, arsenic, mercury and aluminum is still detectable in many PCPs [8]. Following dermal uptake, inhalation or ingestion, compounds may reduce reproductive function through either direct damage to testicular tissue or via endocrine disruption [5,9,10]. The mechanisms for disruption include weak agonism or antagonism to estrogen or androgen receptor activity evident through in vitro and in vivo testing [5]. Though extensive animal studies warn of potential toxicity, knowledge of adverse reproductive effects of PCPs in humans is lacking [5].

Current PCP regulations are widely based on assessment of single product exposures [11]. In reality, consumers often co-use multiple products. Hence, their aggregated exposure may exceed the intended margins of safety for numerous chemicals in the products [12]. Adding to this complexity, consumer habits vary according to age, sex, ethnicity, educational level, skin type, geographical and cultural settings [12,13,14]. As the boundaries of beauty and gender are constantly challenged by modern society, male acceptance of and adaptation to routines previously practiced exclusively by women are rising [6]. Refining our knowledge of the actual use of PCPs in young men may, therefore, improve our options for qualified risk assessment and protection through regulation. Our aims in this study were, therefore, to assess the extent of use and co-use of PCPs and examine potential associations between aggregated exposure and semen quality in a population of young Danish men. Fragrance and flavor components are often protected trade secrets and, therefore, not declared individually for PCPs [15]. As fragranced PCPs may contain higher levels of especially endocrine disrupting chemicals (EDCs), we specifically assessed associations for the use of fragranced PCPs [16,17].

## 2. Materials and Methods

### 2.1. The FEPOS Cohort

The Fetal Programming of Semen Quality (FEPOS) cohort was established with the purpose of identifying potential causes for male infertility throughout the life course to provide options for the improvement of male reproductive health [18]. In brief, young men were sampled from pregnancies included in the Danish National Birth Cohort (DNBC) [19]. The DNBC contains nationwide information on roughly 100,000 pregnancies in Denmark in the period 1996–2002 with a participation rate at enrollment of approximately 60% of the invited women [20,21]. Detailed descriptions of maternal and fetal exposures are available from four pre- and post-natal computer-assisted telephone interviews and gestational blood sampling [19]. Men were considered eligible for inclusion in our FEPOS cohort if their mothers had completed both blood sampling and the two computer-assisted telephone interviews conducted around gestational weeks 16 and 30 [18]. In addition, participating men had to be at least 18 years and 9 months of age upon invitation and live within reasonable distance of one of the study clinics in either Copenhagen or Aarhus [18]. Criteria for exclusion were a medical history of chemotherapy treatment, sterilization or orchiectomy procedures.

Participants were recruited through a secure digital mailbox system (e-Boks) from March 2017 to December 2019 [18]. Each participant answered a comprehensive online questionnaire and provided a semen sample as part of a thorough clinical examination. The overall response rate was 19%. Through subsequent linkage of personal identification numbers, further medical history was obtained from the Danish National Patient Register (DNPR) [22,23]. This register contains virtually complete records of diagnoses from all Danish hospital admissions since 1977 and outpatient visits since 1995 [23].

While 1058 young men were enrolled in the FEPOS cohort at the time of this cross-sectional study, 12 had to be excluded from analyses for not providing semen samples (*n* = 8) or information on PCP usage (*n* = 4).

### 2.2. PCP Exposure

Measures of PCP exposure were derived from the online questionnaire answered by each participant prior to the clinical examination. Based on the extent of product use identified in the existing literature, 12 PCPs were selected for assessment representing several main categories of commonly used products (Table 1) [12,13,14,24]. For each product type, current user status was assessed based on a frequency of use of more than once a week. The available questionnaire information is presented in Table 1. The categories for answers (no = 0/yes, without fragrance = 1/yes, with fragrance = 1) were mutually exclusive and summarized across products into both an overall aggregated score of use and an additional aggregated score covering only fragranced products (range, 0–12). Exposure contrasts were examined with a categorical split for every three PCPs used. The two highest exposure strata were collapsed in the analyses due to low numbers of participants (low ≤ 3 PCPs, medium = 4–6 PCPs, and high ≥ 7 PCPs). As the number of fragranced products used was lower, this measure was split for every two fragranced PCPs. Here, exposure strata with more than four products were collapsed in analyses (low ≤ 2, medium = 3–4 and high ≥ 5).

### 2.3. Outcome Measures

Semen samples were collected by masturbation after a recommended abstinence period of 2–4 days since last ejaculation. In respect of varying needs for privacy, participants were able to choose between collection at home or at a study clinic. For home collection, participants received a sterile, polypropylene sample kit by mail with specific instructions for timely and temperate transportation to the nearest study clinic [18]. Following delivery of the semen sample to or at the clinic, analyses were initiated immediately (83% within 1 h and 99% within 2 h of ejaculation) with recording of specific abstinence time, potential spillage and measurement of semen volume by weight (1 g = 1 mL). A comprehensive assessment of motility, total sperm count, sperm concentration and morphology was performed in complete accordance with the recommendations from the World Health Organization (WHO) 2010 by a specially trained medical laboratory technician (one at each clinic) [18,25]. Our specific procedures have been described in further detail previously [18]. In order to ensure adequate precision in these analyses, a quality control program was established with both systematic internal comparisons and an external reference laboratory at the Reproductive Medicine Centre in Malmö, Sweden. Coefficients of variation (CVs) for comparisons between FEPOS and the reference laboratory results were acceptable for all selected measures (i.e., FEPOS and reference CVs were 18.4% and 17.6%, respectively, for sperm concentration and 12.7% and 38.6%, respectively, for sperm motility in January 2018 (based on five samples) [18].

### 2.4. Statistical Analyses

Initially, we performed descriptive analyses to provide basic information about available variables by level of exposure to PCPs. The extent of use and co-use of PCPs were examined through simple percentage distributions and calculation of phi coefficients for various combinations of products.

In order to assess a potential association between aggregated exposure to PCPs (overall and fragranced products only) and measures of semen quality, we applied a negative binomial regression model. The non-normal distribution of data and presence of zero values favored the best fit with this model. Estimates were presented as the percentage difference between the low and the medium or high PCP exposure groups with all other variables held constant. In analyses of semen volume and total sperm count, participants reporting semen spillage were excluded (*n* = 179). Similarly, participants with azoospermia were excluded from analyses of motility and morphology (*n* = 15). Finally, data on morphology were unavailable for 5 participants and these were excluded from morphology analyses.

Regression estimates were adjusted for several potential confounders selected a priori based on evidence in the existing literature and directed acyclic graphs (DAGs) [26]. Categorical covariates included alcohol consumption (never or former, <1 time/week, 1–2 times/week, ≥3 times/week), smoking and vaping separately (never, former or occasional, daily or weekly), weekly exercise (none, 1–3 times, ≥4 times), body mass index (continuous, kg/m^2^), ability to grow facial hair sufficient for regular shaving (no, yes), acne (no, yes) and season (spring, summer, fall, winter). Family occupational status during the prenatal period was included based on the highest grade of occupation, maternal or paternal whichever was highest, following the Danish International Classification of Occupations (DISCO-88) and the International Standard Classification of Education (ISCED) (high grade professional, low grade professional, skilled worker, unskilled worker, student, economically inactive, unclassifiable) [27]. In analyses for fragranced PCPs, overall PCP usage was also included as a covariate. While the levels of EDCs may be lower in PCPs without fragrance, usage still contributes to the overall load of these chemicals. Considering the narrow age interval (range 18–21 years) of participants, age was not included in the adjusted analyses.

A number of additional, important factors were included in adjustments to increase precision of resulting estimates: abstinence time (continuous, days), sampling site (home, clinic), place of analyses (Copenhagen, Aarhus) and for motility also time from ejaculation to analyses (continuous, min). Finally, we adjusted for current or previous urogenital disorders potentially associated with semen quality either self-reported in the questionnaires or retrieved from records in the DNPR. The following disorders with corresponding international classification of diseases (ICD) 10 codes were included in adjustments: orchitis and epididymitis N45–N45.9 and N51.1, hydrocele N43–N43.3, varicocele I86.1, torsion of testis N44–N44.9E, cryptorchidism Q53–Q53.9, hypospadias Q54–Q54.9 and phimosis N47. Nine participants with missing data for covariates were excluded in the adjusted analyses.

Finally, we performed a sensitivity analysis excluding all participants with azoospermia from the regression models, as the etiology and risk factors associated with this type of infertility may differ from those influencing the remaining reproductive functioning spectrum [28].

All statistical analyses were conducted using Stata V. 15 (StataCorp, College Station, TX, USA).

### 2.5. Ethics

The study was conducted in accordance with the principles of the Declaration of Helsinki [29]. Ethical approvals were obtained from the Regional Scientific Ethical Committee for Copenhagen and Frederiksberg (VEK) (no. H-16015857, approved 14 September 2016), the Danish Data Protection Agency (No. 2012-58-0004, approved 7 September 2016), and the Steering Committee of the DNBC (No. 2016-08, approved 13 September 2016). All participants gave written informed consent prior to their inclusion in the study.

## 3. Results

All of the 1046 participants were exposed to at least one of the 12 selected PCPs more than once a week. The mean number (SD) of PCPs in current use was 5.3 (2.0). For fragranced PCPs, 962 (92%) participants reported current, weekly usage. Characteristics of the FEPOS cohort stratified by aggregated PCP usage are presented in Table 2. According to the basic information on participating men, their mothers and various outcome variables listed here, there were no substantial differences between the low, medium and high exposure groups. However, men in the high PCP exposure category were slightly more likely to be current smokers, exercise regularly and have sparse facial hair (Table 2).

**Figure 1:**
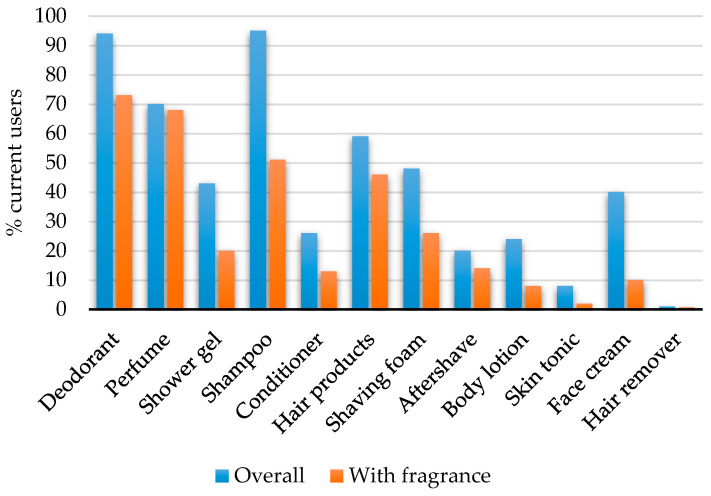
Percentages of current users of personal care products among the 1046 men in the FEPOS cohort.

For the individual PCPs, the highest prevalence of use was observed for shampoo (95%) and deodorant (94%) (Figure 1). The share of participants using fragranced PCPs was higher for the majority of the products with the exception of shower gel, face cream, body lotion and skin tonic (Figure 1). In the analysis of co-use, the obtained phi coefficients were generally weak (Table 3). The strongest associations were identified between use of perfume and hair products (φ = 0.29, *p* < 0.05), followed by use of shaving foam and aftershave (φ = 0.27, *p* < 0.05), and use of body lotion and face cream (φ = 0.26, *p* < 0.05).

Semen characteristics according to aggregated PCP usage are presented in Table 4. We found little association between aggregated overall PCP exposure and semen quality in both crude and adjusted negative binomial regression models for semen volume, total sperm count, sperm concentration, motility or morphology (Table 5). Limiting the exposure variable to fragranced PCPs, little association appeared for the semen quality outcomes. Further, the complete exclusion of men with azoospermia in our sensitivity analysis added no substantial changes to our results (data not shown).

The following covariates were included in all adjusted analyses: alcohol consumption, smoking, vaping, exercise, body mass index, ability to grow facial hair, acne, family occupational status, urogenital disorders, season of sampling, sampling site, place of analysis, abstinence time and time from ejaculation to analysis (only motility). In adjusted analyses for fragranced PCPs, overall PCP usage was included as a covariate. Men with missing data for body mass index, sampling site, time from ejaculation to analysis or abstinence time were excluded from adjusted analyses.

## 4. Discussion

In this first study on self-reported aggregated PCP exposure and male reproductive health, we assessed the extent of product use and the potential associations with measures of semen quality in a large cohort of young men. Despite prevalent use of multiple products, we found little indication of an association with semen quality. While these findings may appear as a reassurance of safety, several issues still need to be addressed.

Due to substantial variations in exposure assessment methods and study populations, the observed levels of and combinations in PCP usage are not directly comparable to any in previous studies [12,13,14,24,30]. The prevalent use of general hygiene products and highly individual approach to adding and combining other care products in our Danish cohort is, however, generally corroborated by the existing literature [13,14,30]. Discrepancies in the observed prevalence of PCP usage between this study and previous studies may widely be attributed to the young age of our participating men [13,14,30]. Thus, the development of especially facial hair is far from fully completed in early adulthood and may not require regular shaving and use of beard or shaving products [31].

The young men in our study widely favored the use of fragranced PCPs. Product declarations often lack the transparency necessary to present an actual informed choice based on contents. Consumers may, therefore, be inclined to let other factors influence their personal care habits (i.e., relationship status, brand name and loyalty, price, income, celebrity or peer endorsement, self-image, sustainability, ethics, promotion, availability and quality) [17,32]. Despite potentially higher levels of EDCs in fragranced PCPs, we found little association between exposure to these products and semen quality [17].

In the extensive epidemiological literature on associations between exposure to specific EDCs present in PCPs and semen quality, results are widely inconsistent and mostly fail to reproduce the effects observed in animal models [5,33,34,35,36,37,38,39,40,41,42]. While a small number of previous studies have indicated a connection between measured levels of parabens, phthalates, glycol ethers, benzophenone UV filters, triclosan and bisphenol A, and semen characteristics, the overall evidence remains limited for these compounds [43]. Endocrine disruption may, however, not be attributable to the presence of single compounds and rather amount from the load of many different chemicals present in relatively low concentrations [43]. Cosmetic chemicals are absorbed slowly across skin in humans compared to rodents and the concentrations achieved in tissues following ordinary use of PCPs may also be too low to inflict clinically relevant damage to male reproduction [44]. Especially estrogen receptors display promiscuity through the ability to bind a range of different compounds with low affinity, but actual, biological effects of these ligands require high doses [5]. Aside from endocrine disruption, reproductive tissues can also be damaged directly by several heavy metals potentially present in PCPs [8]. High concentrations of metals have mainly been detected in pigmented color cosmetics (i.e., lipstick and eye shadow), herbal cosmetics and skin-lightening creams, which have limited use among Scandinavian men [8,45]. Justifying our continued concerns, individual susceptibility to the damaging effects of PCPs may, however, largely depend on genetic modifications and the specific timing of exposure—during fetal life, childhood, adolescence and adulthood [46,47]. In addition, potential damage to sperm DNA integrity and epigenetic changes were not evaluated in this study [3]. Finally, male use of PCPs is increasing rapidly worldwide, and continued efforts to assess aggregated consumer exposures and potential adverse health effects are, therefore, warranted [48]. While authorities are cooperating internationally to achieve regulatory convergence and ensure consumer safety across the global cosmetics industry, restrictions for PCPs and the actual enforcement of these still vary from country to country [49]. A complete evaluation of risks associated with PCP exposure will, therefore, require consideration of differences in national levels of consumer protection.

There were several important limitations in our study. First, our assessment of PCP exposure was rather crude. Considering the extent of the full questionnaire answered by the participants in this study, limitations, especially with respect to the number of PCPs examined, were necessary and may have led to an underestimation of actual exposures. Thus, exposures related to oral hygiene products (toothpaste, mouthwash, mouth spray), lip balm or tanning products (sunscreen, after sun and bronzers) were not accounted for [14,30]. While oral hygiene products were omitted from assessment based on their virtually complete integration in everyone’s daily routines, tanning products were not listed among our current weekly usage items due to the irregularity of use with highly sporadic sun exposures in Denmark [24,50]. Previous studies on consumer exposures have included up to 150 different types of PCPs with potentially greater reflection of the full range and contrast in usage [12].

While we accounted for contributions from co-use of and fragrance in PCPs, additional factors such as the way of use (leave-on, rinse-off), type of product (spray, roll-on), ventilation (if airborne), application area, amount and frequency also affect exposure levels and hence the potential systemic exposure doses (SEDs) for chemical contents [13,14]. Finally, the potential presence of other environmental sources of chemicals may have obscured our results (i.e., home care products along with contaminated food and beverages, pollutants in air, dust and water) [43]. Previous studies have, however, confirmed the validity of self-reported PCP usage as a proxy of systemic exposure to several EDCs through comparisons with direct measurements in body fluids [16,51].

Outcomes were measured directly in biological samples using standardized, state-of-the-art analysis techniques with both internal and external quality control setups. Even in such settings, semen quality parameters often present substantial intraindividual variability and reliability of measures may be increased somewhat through repeated sampling [47]. Thus, errors from misclassification of exposures or outcomes in our study cannot be ruled out.

We were able to adjust for a range of potential confounders covering both pre- and postnatal exposures. However, the use of PCPs reflects capacity for self-care and exposure can, to some extent, be interpreted as a proxy of other aspects of health behavior. Thus, confounding from unknown or unmeasured factors or residual confounding may have influenced our results. In addition, the overall response rate in our study was low (19%). Participation rates below 30% are not unusual in studies assessing sperm characteristics and may indicate bias from selection among participants [47]. Our participants were unaware of the specific exposures at interest and likely also their own outcome status at enrollment, minimizing any bias from selection on these parameters. The participants in our offspring cohort represent a rather narrow age spectrum of primarily Caucasian men living in urban areas. They were sampled from the Danish National Birth Cohort with an underrepresentation of mothers of single status and/or with lower socioeconomic position [20]. Thus, we cannot exclude that selection bias may have affected our results.

A final important limitation in our study was the lack of certainty in temporality between exposure and outcome. The median time between questionnaire completion (PCP exposure assessment) and semen collection was 26 days among the young men in the FEPOS cohort. Impairment of spermatogenesis typically manifests in the semen ejaculate approximately 9 weeks after a harmful exposure (the duration of spermatogenesis) [47]. However, the specific latency with which a potentially harmful exposure can be detected in the ejaculate depends on the affected developmental stage of spermatogenesis [52]. Potential variations in PCP usage over time were not reflected in our cross-sectional study design focusing exclusively on current exposure status. While many adults have relatively fixed personal care routines, younger individuals are both more likely to initiate changes in their habits and less likely to maintain them [53].

The major strength in this study was the use of a large birth cohort with extensive questionnaire information from both the adult participants themselves and their mothers during pregnancy as well as nationwide health register data.

## 5. Conclusions

Despite prevalent use of multiple PCPs among the young men in this study, our findings indicate little association between aggregated exposure to PCPs and semen quality. Exposure assessment was based on the overall number of PCPs in current and frequent use. Thus, reproductive toxicity from individual, specific PCPs cannot be excluded at this point. With an expected global increase in male PCP usage, continued efforts to assess consumer exposures and potential health effects are recommended.

## Figures and Tables

**Table 1:** Items used to assess aggregated PCP usage in the Fetal Programming of Semen Quality (FEPOS) cohort.

Do You Use the Following Products Every Day or Several Times a Week?
Answer Options: No/Yes, Without Fragrance (i.e., Nordic Swan Ecolabel)/Yes, with Fragrance
Deodorant
Perfume
Shower gel
Shampoo
Conditioner
Hair products (gel, spray, etc.)
Shaving foam
Aftershave
Body lotion
Skin tonic
Face cream
Hair remover

PCP, personal care product.

**Table 2:** Characteristics of the FEPOS cohort stratified by aggregated PCP usage.

Characteristics	Overall PCP Usage		
	Low	Medium	High
	(≤3 PCPs)	(4–6 PCPs)	(≥7 PCPs)
**Men**			
Total men, *n*	203	578	265
BMI, mean (SD)	22.1 (3.1)	22.6 (3.6)	22.5 (3.0)
Alcohol ≥ once a week, *n* (%)	97 (48)	316 (55)	151 (57)
Current smoking, *n* (%)	64 (32)	220 (38)	129 (49)
Current vaping, *n* (%)	21 (10)	37 (6)	17 (6)
Regular exercise, *n* (%)	152 (75)	479 (83)	233 (88)
Sparse facial hair, *n* (%) ^1^	157(77)	464 (80)	230 (87)
Acne, *n* (%)	66 (33)	221 (38)	103 (39)
Urogenital disorders, *n* (%) ^2^	36 (18)	121 (21)	55 (21)
**Mothers**			
Age at birth of son, mean (SD)	30.1 (4.0)	30.6 (4.1)	30.5 (4.4)
Smoking during pregnancy, *n* (%)	45 (22)	132 (23)	66 (25)
Pre-pregnancy BMI, mean (SD)	22.6 (3.8)	22.9 (3.6)	22.7 (3.5)
High family occupational status, *n* (%) ^3^	69 (34)	192 (33)	93 (35)
**Semen sample parameters**			
Days of abstinence, mean (SD)	2.5 (2.0)	2.3 (1.4)	2.2 (1.3)
Spillage, yes *n* (%)	33 (16)	96 (17)	50 (19)
Minutes from ejaculation to analysis, mean (SD)	50 (19.9)	49 (19.3)	52 (19.1)
Sampling site, clinic *n* (%)	176 (87)	504 (87)	224 (85)
Place of analysis, Copenhagen *n* (%)	150 (74)	442 (76)	230 (87)

PCP, personal care product; BMI, body mass index. ^1^ Facial hair insufficient for regular shaving. ^2^ Current or previous urogenital disorders potentially associated with semen quality. ^3^ Based on the highest grade of maternal and paternal occupation during pregnancy.

**Table 3:** Phi coefficients for co-use of PCPs among the 1046 men in the FEPOS cohort.

**Item**	**General Hygiene**			**Hair Care**		
	**Deodorant**	**Perfume**	**Shower Gel**	**Shampoo**	**Conditioner**	**Hair Products**
**General hygiene**						
Deodorant						
Perfume	**0.09**					
Shower gel	**0.12**	**0.09**				
**Hair Care**						
Shampoo	0	0.03	−0.07			
Conditioner	0	**0.15**	**0.1**	**0.09**		
Hair products	**0.08**	**0.29**	0.05	**0.09**	0.05	
**Shaving products**						
Shaving foam	0.03	**0.17**	0.05	0.05	0.04	**0.11**
Aftershave	0.05	**0.14**	**0.14**	−0.03	**0.11**	0.02
**Skin care**						
Body lotion	0.01	**0.13**	**0.1**	−0.01	**0.14**	**0.12**
Skin tonic	0.03	**0.06**	**0.1**	−0.00	0.06	0.06
Face cream	0.06	**0.2**	**0.09**	**−0.09**	**0.12**	**0.18**
**Other**						
Hair remover	0.03	0.03	0.04	−0.01	**0.08**	−0.01
**Item**	**Shaving Product**		**Skin Care**			
	**Shaving Foam**	**Aftershave**	**Body Lotion**	**Skin Tonic**	**Face Cream**	
Aftershave	**0.27**					
**Skin care**						
Body lotion	0.05	**0.08**				
Skin tonic	**0.08**	**0.14**	**0.08**			
Face cream	**0.14**	**0.17**	**0.26**	**0.21**		
**Other**						
Hair remover	0.03	0.06	**0.07**	**0.12**	0.02	

PCP, personal care product. Results significant at *p* 0.05 level are marked in bold.

**Table 4:** Semen characteristics for the 1046 men in the FEPOS cohort stratified by aggregated PCP usage.

Parameter	N ^1^	Overall PCP Usage					
		Low		Medium		High	
		(≤3 PCPs)		(4–6 PCPs)		(≥7 PCPs)	
		Md	P_25%_, P_75%_	Md	P_25%_, P_75%_	Md	P_25%_, P_75%_
**Sperm concentration (10^6^/mL)**	1046	38	18, 73	40	19, 76	38	20, 65
**Total sperm count (10^6^)**	867	105	41, 204	99	45, 202	106	52, 196
**Semen volume (mL)**	867	2.9	2.0, 3.8	2.6	1.8, 3.6	2.7	2.0, 3.7
**Progressive motility (%)**	1031	64	52, 72	63	52, 74	63	53, 74
**Morphology, normal (%)**	1026	7	4, 10	6	3, 10	6	2, 10

PCP, personal care product; Md, median. ^1^ In analyses of total sperm count and semen volume, men reporting spillage were excluded (*n* = 179). Similarly, azoospermic men were excluded from analyses of motility and morphology (*n* = 15). Men with unavailable morphology data were excluded from morphology analyses (*n* = 5).

**Table 5:** Negative binomial regression analyses of semen characteristics in relation to aggregated PCP usage among the 1046 men in the FEPOS cohort.

**Characteristics**	**Model ^1^**	**N**	**Overall PCP Usage**		
			**Low**	**Medium**	**High**
			**(≤3 PCPs)**	**(4–6 PCPs)**	**(≥7 PCPs)**
			**Reference**	**% Difference (95% CI)**	**% Difference (95% CI)**
**Sperm concentration (10^6^/mL)**	Crude	1046	0	2 (−11, 18)	−8 (−22, 8)
	Adjusted	1037	0	9 (−5, 26)	−2 (−17, 15)
**Total sperm count (10^6^)**	Crude	867	0	−3 (−18, 15)	−4 (−21, 17)
	Adjusted	858	0	−4 (−18, 13)	−3 (−20, 16)
**Semen volume (mL)**	Crude	866	0	−8 (−17, 2)	−3 (−14, 9)
	Adjusted	857	0	−4 (−14, 7)	0 (−12, 13)
**Motility, 100-progressive (%)**	Crude	1031	0	0 (−4, 7)	0 (−7, 8)
	Adjusted	1016	0	2 (−5, 8)	3 (−4, 11)
**Morphology, normal (%)**	Crude	1026	0	−8 (−19, 4)	−8 (−20, 6)
	Adjusted	1017	0	−7 (−18, 5)	−8 (−20, 7)
**Characteristics Model ^1^ N**			**Fragranced PCP Usage**		
			**Low**	**Medium**	**High**
			**(≤2 PCPs)**	**(3–4 PCPs)**	**(≥5 PCPs)**
			**Reference**	**% Difference (95% CI)**	**% Difference (95% CI)**
**Sperm concentration (10^6^/mL)**	Crude	1046	0	−5 (−16, 8)	−6 (−18, 9)
	Adjusted	1037	0	0 (−12, 14)	6 (−9, 24)
**Total sperm count (10^6^)**	Crude	867	0	1 (−13, 17)	−6 (−20, 11)
	Adjusted	858	0	5 (−10, 22)	7 (−11, 28)
**Semen volume (mL)**	Crude	866	0	1 (−8, 10)	−4 (−14, 6)
	Adjusted	857	0	5 (−5, 15)	1 (−10, 14)
**Motility, 100-progressive (%)**	Crude	1031	0	0 (−6, 5)	−4 (−10, 2)
	Adjusted	1016	0	0 (−6, 6)	−4 (−11, 3)
**Morphology, normal (%)**	Crude	1026	0	−4 (−14, 7)	−3 (−14, 9)
	Adjusted	1017	0	−1 (−12, 11)	3 (−11, 18)

PCP, personal care product. ^1^ In analyses of total sperm count and semen volume, men reporting spillage were excluded (*n* = 179). Similarly, azoospermic men were excluded from analyses of motility and morphology (*n* = 15). Men with unavailable morphology data were excluded from morphology analyses (*n* = 5).
